# Comparison of photographic and visual assessment of occlusal caries with histology as the reference standard

**DOI:** 10.1186/1472-6831-12-10

**Published:** 2012-04-27

**Authors:** Uriana Boye, Tanya Walsh, Iain A Pretty, Martin Tickle

**Affiliations:** 1 The Oral Health Unit, School of Dentistry, University of Manchester, Manchester Academic Health Sciences Centre, Manchester, UK; 2 The Cochrane Oral Health Group, University of Manchester, Manchester Academic Health Sciences Centre, Manchester, UK; 3 The Dental Health Unit, 3A Skelton House, Lloyd Street North, Manchester, M15 6SH, UK

## Abstract

**Background:**

The purpose of this study was to compare diagnostic performance for the detection of caries using photographs with an established visual examination method and histological sections as the reference standard.

**Methods:**

50 extracted permanent teeth were assessed for the presence of occlusal caries by 9 examiners using two methods; traditional visual examination developed by BASCD and photographs produced by an intra-oral camera. For both methods, diagnoses were made at “caries into dentine” level. The teeth were histologically sectioned and the diagnostic decisions using visual and photographic assessment were compared to the histological reference standard. Inter- and intra- examiner reliability for the methods was assessed and weighted kappa values were calculated.

**Results:**

The visual examination method had a median sensitivity value of 65.6% and a median specificity value of 82.4%. The photographic assessments method had a median sensitivity of 81.3% and a median specificity of 82.4%.

**Conclusions:**

The photographic assessments method had a higher sensitivity for caries detection than the visual examination. The two methods had comparable specificities and good intra- and inter- examiner reliability.

## Background

Dental caries is still the principal challenge that occupies the efforts of clinical and public health dentists alike. Whether in the field of caries research, dental education and dental epidemiology or in the clinical decision making in dental practices, the appropriate means of caries detection and measurement is required. This has led to proliferation of literature about how best to detect and diagnose dental caries in various settings [1]. The most common and traditional method is by a visual inspection of the tooth surfaces. Great progress has been made in the development of novel techniques and technologies that aid the detection of dental caries. These caries detection aids aspire to increase the sensitivity of visual caries detection as well as maintaining a good level of specificity [2]. The majority of these systems were validated using visual caries determination methods [3-5]. The need for clinically reliable caries detection methods has however led to the development and refinement of visual systems such as the ICDAS (International Caries Detection and Assessment System) and the Universal Visual Scoring System (UniViSS) [6,7].

Dental surveillance surveys and large epidemiological studies have traditionally depended on the use of visual dental examination techniques as the caries detection method. This is because for dental public health purposes, visual determination is simple, requires low technology and is easy to administer. This method of caries detection is however not appropriate in comparative studies where examiners collecting caries information need to be “blind” to various attributes of the different populations; for example the residential status of participants in the evaluation of water fluoridation schemes or to the allocation of participants in randomized control trials. Other caries detection methods such as radiographs, or more innovative techniques/technologies such as Quantitative Light-induced Fluorescence (QLF), fibre-optic trans-illumination (FOTI) and electronic caries monitoring (ECM) [8] will not be suitable to use in such studies for a variety of reasons.

The use of radiographs to ensure examiner “blinding” is an unviable proposition as it is fraught with ethical dilemmas in terms of justifiable risks from exposure to ionizing radiation and problems with validity in detecting occlusal caries [9]. Although dental caries and enamel fluorosis present as different lesions, it may be difficult to use the QLF techniques that are commercially available to make a distinction [10]. QLF is more suited to laboratory based research and clinical work involving precise measurement of changes in mineralization of tooth-tissue. ECM is a very sensitive caries detection method which can be affected by factors such as the presence of water, ambient light, and tooth temperature [11].

As digital imaging fibre-optic trans-illumination (DiFOTI) produces images that can be stored, it could be considered for ensuring examiner “blinding”. It is however very cumbersome to handle and time consuming to use. It also requires considerable amount of training to achieve the level of competence needed for it to be used as a caries detection method [8]. As such, none of the so called “novel” methods for caries detection appear appropriate for use in epidemiological studies where blinding is required.

However, a simple and economical method of “blinding” may be for examiners to inspect photographs of participants’ teeth rather than examine the subjects visually.

Photographic images have been used in dentistry in a variety of ways [12-14] and intra- and extra- oral cameras have evolved rapidly over recent years with a commensurate decrease in cost and complexity of use. With the advancement in technology, there are various intra-oral cameras now in use in clinical dental practice. There are however very few studies in the literature that have investigated the use of intra-oral images and caries diagnosis. In a study by Elfrink et al [15], intra-oral photographs were used to score caries and hypo-mineralization on primary molars in a clinical setting and the results suggest that intra-oral photographs may be used in clinical practice and large epidemiological studies with some degree of confidence. The main method of caries determination in the UK National Health Service epidemiological surveys is via a visual examination method developed and described by the British Association for the study of Community Dentistry (BASCD) [16-18]. Before intra-oral photographs can be recommended for use in epidemiological studies their performance must be assessed against the established BASCD visual examination method and the reference standard for caries diagnosis of histological section.

### Aims

The purpose of this study was to compare diagnostic performance for the detection of caries into dentine of photographs with an established visual examination method and histological section as the reference standard. The following hypotheses will be tested to determine if:

1. There is significant difference in visual examination scores for the extracted teeth recorded by a group of examiners (to test inter-examiner reliability for the visual examination)

2. There is significant difference in visual examination scores for the extracted teeth recorded by the same examiner on two different occasions (to test intra-examiner reliability for the visual examination method)

3. There is a significant difference in photographic assessments of the extracted teeth viewed by a group of assessors (to test inter-examiner reliability for the photographic assessments)

4. There is a significant difference in assessments scores of photographs of the extracted teeth viewed by the same assessor on two different occasions (to test intra-examiner reliability for the photographic assessments)

5. There is a significant difference in recorded dental caries between the visual, photographic and histological methods of detecting caries at “the caries into dentine” level.

## Methods

Prior to undertaking the study, ethical approval was granted by the University of Manchester Committee on Ethics of Research on Human Beings (Reference Number 06306). Permanent extracted teeth, supplied by the University Of Indianapolis School Of Dentistry were used for the study. Patients from who the teeth were obtained gave their consent for the teeth to be used in any non-DNA dental research. Teeth with lesions other than caries and teeth with restorations and/or fissure sealants were excluded from the study. The teeth were subsequently anonymised. The study was conducted to the Helsinki Declaration and local legislation as determined by the ethics committee whose approval was gained.

**Figure 1:**
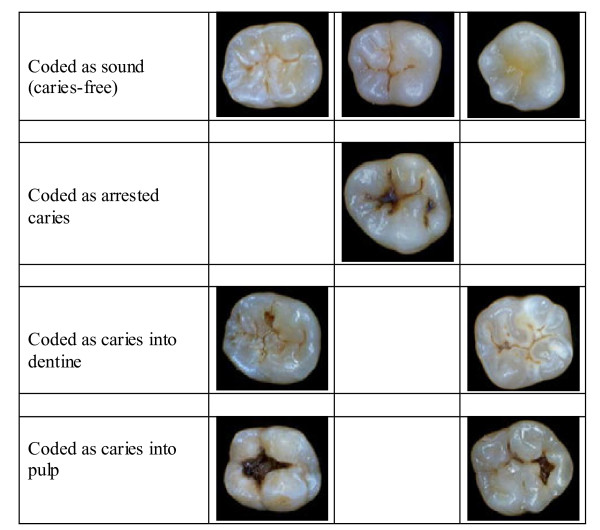
Example of photos of the extracted teeth showing the various codes.

Fifty permanent extracted teeth, 32 molars and 18 premolars, varying from sound to grossly carious teeth were used for this study. The teeth were examined visually for caries (without probing) using the method developed and described by BASCD [16]. The BASCD codes used for scoring, classified teeth as being sound (caries-free), having arrested caries, having caries into dentine or having caries extending into the pulp (Figure 1). The teeth were also photographed using an intra-oral camera and the obtained images assessed for caries using the same BASCD codes as were used for the visual examination. The teeth were then sectioned for a histological assessment to detect the presence of dental caries.

### Visual examination

Nine examiners trained and calibrated to the BASCD caries examination protocol as members of the team who undertake the UK National Epidemiological Surveys convened to examine the extracted teeth visually for caries. They examined the teeth using the criteria and protocol developed by BASCD. Each examiner assessed the teeth on two separate occasions to test intra-examiner reliability.

The teeth were stored in thymol to prevent microbial contamination. Prior to the examination, two sets of randomly generated identity numbers (ID) were assigned to each tooth, one set for the first exam and the other set for the second examination. For the visual examination each tooth was placed in water within a dappen pot labelled with its assigned ID. The teeth were examined using Daray X100 Lamp with Pivot D desk mount (Daray Healthcare Products® Swadlincote, Derbyshire) as the source of light. Each tooth was dried with cotton wool rolls prior to the examination. Caries was diagnosed visually at the ‘caries into dentine’ level (enamel only caries was not recorded). Only the occlusal surfaces of the teeth were assessed and the assigned BASCD score recorded onto a paper pro-forma. The second examination was conducted 1 hour after the first.

### Photographic procedure and assessment

Each tooth was photographed using an intra-oral camera, the Sopro 717 (The Acteon Group® Eaton Socon, Cambridgeshire), which has an integral LED light source. The teeth were dried using cotton wool rolls prior to being photographed. Each tooth was held in place on an adjustable mount, the level of which was raised or lowered relative to the camera to obtain the best occlusal view photograph. The camera was held by a clamp in a fixed position pointing downwards onto the tooth surface. The digital image of each tooth generated was saved under a file name which was the same as the identity number allocated to that tooth. The photographs were presented as a Microsoft PowerPoint (2003 version) slide show for assessment.

The same 9 examiners who had examined the extracted teeth visually assessed the PowerPoint presentation of the photographs on two separate occasions. On the first occasion, the examiners convened to view the slide show of the teeth. This was to ensure that each examiner assessed the photographs under the same physical conditions. The examiners were seated behind tables and each had a view of one common screen that was 2.5 metres away in a room lit by ambient daylight. The PowerPoint slide show of the teeth was projected on to the screen. Each photograph remained on the screen for 15 seconds. Just as for the visual examination, only the occlusal surfaces of the teeth were assessed for caries using BASCD criteria. Caries was diagnosed at the “caries into dentine” level. Each examiner recorded the scores for each tooth onto a paper pro-forma, identical to the one used for the visual examination. The examiners were supervised and did not concur with each other during the process.

For viewing on the second occasion, each examiner was provided with a non-time limited version of the same PowerPoint presentation on a CD ROM. Each examiner viewed the photographs a minimum of 14 days after the first viewing. Each examiner viewed the slide show on either laptops or desktop computer screens at a time of day and room conditions of their choice. The purpose of the second viewing was to compare the caries detection performance when the photographs were viewed under standardized and varying physical conditions. Only the occlusal surfaces of the teeth were scored for caries using BASCD criteria. Caries was again diagnosed at the “caries into dentine” level. The examiners recorded the scores for each tooth onto a paper pro-forma, identical to the one used for the visual examination.

### Histological assessment

**Figure 2:**
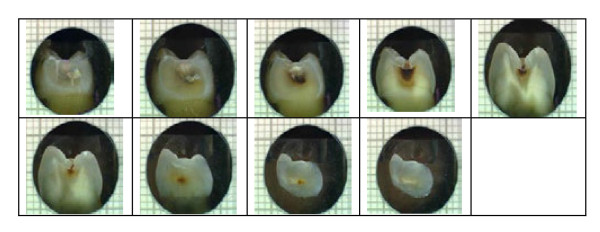
Examples of tooth sectioning procedure.

The extracted teeth were then sectioned for histological assessments. To obtain the histological sections, each tooth was immersed in resin and allowed to set into blocks, with approximately 1.5cm to each side. Each block with an encased tooth was then pressed up against a model grinder, removing thin layers of resin at a time until initial exposure of tooth. The newly exposed tooth surface was polished, dried, and photographed with an extra-oral camera with a ring illuminator. The extra-oral camera with the ring illuminator was pointed upwards, with a small mount on top of it, to house the tooth. This ensured that the tooth was always in focus at the same zoom. The tooth was then returned to the grinder for a while to remove more resin and tooth. The process of alternative grinding and photographing was repeated about 50 times per tooth. The average distance between one photographed cross-section and the next was 0.16mm (Figure 2).

The histological section with the worst level of caries for each tooth was assessed by a trained and standardized examiner. The sections were scored from photographs that were at 10x magnification. The histological assessment was conducted on two separate occasions, one week apart, by the same examiner to test intra-examiner reliability. Sections were scored as sound (caries-free), caries into outer dentine, caries into inner dentine and caries into pulp. These scores were used in the comparative analysis as the reference standard [19].

## Data processing and analysis

Data from the visual examinations, photographic and histological assessments of the extracted teeth were collated for analysis. SPSS® version 15.0 (IBM Company, Chicago) was used to compute weighted kappa scores as a measure of agreement to test intra-examiner reliability for the visual examination and the photographic assessments using the Landis and Koch measurement of observer agreement for categorical data [20]. Inter-examiner reliability was also assessed to test the measure of agreement within the group for both the visual examinations and the photographic assessments using Stata® statistical software version 10 (Stata Corporation Texas).

McNemar test (p<0.05) was performed to compare the performance of the visual and photographic methods using SPSS® version 15.0 (IBM Company, Chicago). SPSS® version 15.0 (IBM Company, Chicago) was also used to compute sensitivity and specificity of the visual examinations using the first histological assessments as the reference standard and sensitivity and specificity of the photographic examinations using histological assessments as the reference standard. For these analyses, the teeth were grouped as sound or carious. All the teeth scored as having arrested caries, caries into inner/outer dentine or caries into pulp by the all the examination types were grouped as carious.

## Results

The frequency distribution of the codes/scores allocation to the teeth according to the examination type is presented in Table 1. Table 2 summaries the intra examiner agreement for both the visual examination and photographic assessments. The level of agreement between the first and second visual examination for the individual examiners ranged from substantial agreement to almost perfect agreement (weighted kappa from 0.67 to 0.92 with a median = 0.85) Landis and Koch [20].

**Table 1:** Frequency distribution of tooth scores according to examination method

	***Examination Method***					
	**Histology**		**Mean Visual Scores**		**Mean Photo Scores**	
Tooth Condition	Freq	Percent	Freq	Percent	Freq	Percent
Sound	18	36.7%	26	52.0%	22	44.0%
Caries into Dentine	24	49.0%	22	44.0%	26	52.0%
Caries into pulp	7	14.3%	2	4.0%	2	4.0%
Missing data	1*					
**Total**	50	100%	50	100%	50	100%

^+^ For histology: inner and outer dentine caries was combined into caries into dentine.

* 1 tooth exploded during histological sectioning.

**Table 2:** Intra-examiner reliability for the Visual and Photographic assessment

***Wt kappa to show level of agreement between***			
		**1^st^ and 2^nd^**	1^st^* and 2^nd^**
		**visual exams**	**Photos assessments**
**Examiner**	A	0.87	0.74
	B	0.85	0.59
	C	0.87	0.69
	D	0.79	0.92
	E	0.67	0.59
	F	0.88	0.78
	G	0.74	0.68
	H	0.92	0.75
	I	0.79	0.84
	**Median**	**0.85**	**0.74**

*Photographic assessments projected on screen.

** Photographic assessments on personal computers.

The level of agreement between the first and second photographic assessment for the individual examiners (to compare standardized with varying physical conditions for viewing photographs) ranged from moderate agreement to almost perfect agreement with weighted kappa from 0.59 to 0.92 with a median = 0.74. [20]

The measure of inter-examiner reliability for the visual examinations was a multi-rater kappa of 0.66. This showed there was substantial agreement within the group for the visual scores. The measure of inter-examiner reliability within the group for the photographic assessment was a multi-rater kappa of 0.60. Again this showed a substantial agreement within the group.

McNemar test (p<0.05) calculated to compare the performance of the visual and photographic methods showed no significant difference between the methods.

Sensitivity and specificity as a measure of the diagnostic performance of the visual examination and the photographic assessment methods as compared to the reference standard of histological section assessments are presented in Table 3.

**Table 3:** Sensitivity and Specificity

	***Reference Standard: Histology***			
	**Visual Examination**		**Photographic assessment**	
Examiner	Sensitivity	Specificity	Sensitivity	Specificity
1	62.5%	100.0%	78.1%	100.0%
2	62.5%	82.4%	68.8%	76.5%
3	68.8%	76.5%	78.1%	82.4%
4	71.9%	76.5%	90.6%	75.5%
5	68.8%	70.6%	81.3%	88.2%
6	71.9%	76.5%	84.4%	82.4%
7	62.5%	82.4%	71.9%	76.5%
8	65.6%	94.1%	78.1%	88.2%
9	65.6%	82.4%	78.15	70.6%
**Median**	**65.5%**	**82.4%**	**81.3%**	**82.4%**

## Discussion

The main findings of this study showed substantial intra- and inter-examiner reliability for both the visual and photographic assessments. The median sensitivity and specificity values of the visual examinations and photographic assessments as compared to the gold standard of histology were 65.5% & 82.4% and 81.3.8% & 82.4% respectively. These showed that the photographic assessment method in this study has a caries detection capability that is comparable to that of the BASCD visual examination method.

The difficulty of convening examiners from wide geographical area made a longer washout period for the visual examinations problematic in this study period. It had to be expected that a decision (one of 50) could not be recalled by the examiners after the washout time allowed in this study. In this study caries was only diagnosed when it was determined by the examiners to have reached dentine. This is because the established visual examination method developed by BASCD that was used for the comparisons determines caries at the caries into dentine level as part of its protocol. Although early caries is not accounted for, this method has provided the main epidemiological data on the state of the dental health of the child population in the UK for almost 30 years [21-23]. Any other caries detection method to be used in dental public health studies in the UK should at least be comparable to the BASCD examination in ease of application, reliability and validity.

The detection of caries was restricted to only the occlusal surfaces of the teeth. The purpose of this approach was to minimize the number of photographs per tooth that had to be assessed by the examiners. This may be seen as limiting the external validity of the findings of this study. However majority of carious lesions occur in pits and fissures on occlusal surfaces [24] and moreover as lesions on occlusal surfaces are the most difficult to reliably diagnose [25] the findings of this study will contribute to the available literature. Future research could include caries detection on other tooth surfaces in vivo. Also weighted kappa rather than non-weighted kappa values were computed and used for the reliability comparisons because the caries diagnosis was not just recorded as dichotomous scores (of caries present or absent) but rather as category of scores.

The physical conditions by which photographs are viewed and how they are presented could potentially influence assessment, reducing intra examiner reliability. However we found good agreement between the standardized and non-standardized presentation of the photographs, suggesting that the mode of presentation has little impact on diagnostic decision making from photographs.

Decisions about the introduction of caries prevention strategies such as water fluoridation and the evaluation of such interventions will depend on detecting the presence of the disease at the appropriate level. The requirements of a caries detection tool needed for such a task will not be the same as that required in, for example, general practice to aid management of the early stages of the disease. Examiners collecting data should however do so at a consistent level; the intra examiner reliability for both the visual and photographic methods was high.

Histology/microscopy is used as the reference standard in many comparison and validity studies [26-29]. Some researchers believe that there is tissue loss during sectioning of teeth and thus histology/microscopy cannot be said to be the ultimate reference standard. This may be important in studies concerning very early enamel lesions but not detecting caries into dentine used for dental public health purposes. The two methods in this study were compared to histology as the reference standard to determine their validity. The photographic assessments method had a higher sensitivity (median value 81.3%) for caries detection than the visual examination (median value 65.6%). The two methods however had the same specificity (82.4%) which was high. The photographic assessment method in this study has caries detection capability that is at least comparable to that of the BASCD visual examination method.

As well as the possible advantages concerning blinding in comparative studies, another benefit that the photographic method could provide for dental public health epidemiological research is the archiving of images. This is a concept which is already widely used in “Store and forward” telemedicine [30,31]. The use of intra-oral photographs would allow remote dental examination and screening as piloted by a previous study [32]. In theory, trained dental nurses or other dental care professionals could take the intra-oral photographs for a single examiner to assess them all. The use of this approach in epidemiological studies will eliminate potential errors that could be introduced by using multiple assessors if potential logistical issues of using the system in the field can be mitigated.

Also archived photographic records would allow true comparative public health studies on the same cohort of population or different cohorts based on archived images in future research. This could include future water fluoridation evaluation studies. These could be conducted as prospective cohort studies where examiners assess the photographs of participants in both the test and control groups “blind” to participants’ exposure to water fluoridation thereby reducing the risk of bias.

The findings of this study relate to teeth in in-vitro condition. The next stage in the development of the use of intra oral photographs in dental public health epidemiological studies is to explore their performance in an in-vivo study comparing the two methods.

## Conclusions

In summary, the comparisons in this study showed that the assessments of the photographs as a method of caries detection had a higher sensitivity than visual examination compared to the reference standard of histology. The two methods however had comparable specificities. There was also good intra examiner and inter examiner reliability for the examiners assessing the photographic images.

## Competing interests

None of the authors are aware of any competing interests in the production of this manuscript.

## Authors’ contributions

UB contributed to the protocol, undertook the management of the study, took the photographs and wrote part of the manuscript. TW gave statistical advice, assisted with data analysis and contributed to the manuscript. IAP contributed to the protocol, undertook study monitoring and wrote part of the manuscript. MT contributed to the protocol, undertook study monitoring and wrote part of the manuscript. All authors read and approved the final manuscript.

## Disclaimer

The views and opinions expressed are those of the authors and do not necessarily reflect those of the NHS.

## Pre-publication history

The pre-publication history for this paper can be accessed here:

http://www.biomedcentral.com/1472-6831/12/10/prepub
